# Effectiveness of heat stress interventions among outdoor workers: a protocol paper

**DOI:** 10.3389/fpubh.2024.1477186

**Published:** 2024-10-25

**Authors:** Sajeeth Kumar Sankar, Vijayalakshmi P, Krishnan S, Rekha Shanmugam, Latha Perumal Kamalakkanan, Vidhya Venugopal

**Affiliations:** ^1^Department of Environmental Health Engineering, Faculty of Public Health, Sri Ramachandra Institute of Higher Education and Research, Chennai, India; ^2^Department of Applied Psychology, Faculty of Behavioural and Social Science, Sri Ramachandra Institute of Higher Education and Research, Chennai, India; ^3^Department of Physiology, All India Institute of Medical Sciences, Guwahati, Assam, India

**Keywords:** heat stress, intervention, outdoor workers, physiological heat strain, WRS

## Abstract

**Introduction:**

Heavy work in scorching temperatures can cause dehydration and heat stress, which can lead to a number of heat-related diseases. Heavy work in intense heat without rest or hydration is the main cause. The “Water, Rest, Shade” (WRS) intervention program for outdoor workers in hot weather reduces heat stress.

**Methods:**

This study follows a quasi-experimental design involving 250 outdoor workers from both agriculture and brick kilns. To assess the environmental heat exposure levels, Quest Temp 3 M WBGT Monitor will be used. Tympanic temperature, Heart rate (HR), Sweat rate (SwR), and Urine specific gravity (USG) to assess the physiological responses to heat exposure using standard protocols. Blood samples will be collected to measure serum creatinine and calculate Glomerular filtration rate (GFR), and urine samples to measure pH, leucocytes, proteinuria, and hematuria. Then administer a validated and modified HOTHAPS questionnaire to capture the perception data. After the baseline assessments, Categorize the workers into two groups based on the selection criteria and the participants’ willingness. Then provide a week-long WRS intervention to the intervention group (IG). The non-intervention group will collect the same data without any intervention to assess the efficacy of the intervention by comparing both groups and measuring outcome indicators.

**Expected outcome:**

The study will generate much-needed information to raise awareness of the importance of heat stress prevention for outdoor workers.

**Conclusion:**

This study will demonstrate the effectiveness of an intervention, provide much-needed strategies for reducing heat stress, assess both health impacts and implementation quality, and design comprehensive workplace and labor laws aim to minimise risks to millions of unorganised outdoor workers health.

## 1 Introduction

Over the next few decades, it is anticipated that global temperatures will rise by 0.5 to 1.5°C that threaten all living things, including humans (1). In particular, the working environments are impacted by climate change, posing health risks to millions of workers (2). Workplace heat poses a risk to workers in various outdoor environments, resulting in a range of Heat-Related Illnesses (HRI) that can manifest when the body’s heat absorption and production exceeds its heat dissipation capacity (3). Physically demanding jobs performed in high-temperature environments place individuals at a heightened risk (4).

Heat stress is a condition that can occur due to prolonged exposure to elevated temperatures. When the body’s mechanism for regulating internal temperature begins to struggle, heat stress occurs (5). When a worker is exposed to various factors like metabolic heat, environmental conditions, and clothing, it leads to an accumulation of heat in the body. It leads to internal body temperatures rise as well as decreased productivity and performance (6). And it leads to heat related illness (HRI), such as distress, headaches, syncope, loss of mental awareness, heat stroke (7). When core body temperature (CBT) raises above 42°C can lead to various adverse outcomes, such as harm to major organs and potential death (8).

Over the years, heat waves were responsible for more than 166,000 deaths between 1998 and 2017. The majority of outdoor workers had a low estimated glomerular filtration rate (eGFR), it shows kidney function impairment due to heat exposure (9). In addition to the deaths associated with heat waves, there is also a strong correlation between heat stress and the emergence or worsening of several non-communicable diseases (NCDs). These include chronic kidney disease (CKD), cardiovascular disorders, and respiratory morbidity. India is known for its significant heat events, particularly during the summer season faces a considerable challenge in terms of human health and well-being. These heat events manifest in the form of heat waves, posing significant threats to outdoor workers (5). Based on the available projections, it is anticipated that India could potentially experience a decline in its gross domestic product (GDP) within the range of 2.5 to 4.5% by the year 2030 (10). The decline observed in this particular scenario can be linked to a decrease in labour hours, which can be primarily attributed to the adverse impacts of extreme heat and humidity conditions. It is noteworthy that approximately 40% of the nation’s GDP is associated with occupations that are exposed to high levels of heat-exposed work, and over 90% of the workforce is engaged in informal employment (11).

Informal work is described as work that lacks a formal contract, paid time off, or other benefits (12). Every year, India witnesses a significant rise in temperatures. This situation results in reduced productivity, negative health effects, and economic losses, accompanied by an increase in heat-related fatalities among the workforce (13). The current situation in India highlights a significant issue concerning the impact of rising temperatures on the outdoor labour force (14). Regrettably, there is a lack of a comprehensive action plan or policy in place to protect the well-being of these workers in the face of challenging, hot, and humid working conditions. These individuals often undergo a conventional 8-h workday marked by insufficient breaks, rest intervals, access to sufficient toilets, and water intake (15). The lack of proper protective measures results in adverse health effects; consequently, practical and sufficient regulations, as well as heat stress interventions, are not effectively put into practice (16).

One of the foremost public health strategies involves educating people on preventing heat stress and identifying its early warning signs. Occupational Safety and Health Administration (OSHA) strongly recommends three words: water, rest, and shade intervention to protect workers from heat stress. The Water, Rest, Shade (WRS) intervention has been chosen based on its demonstrated effectiveness in managing heat stress in various occupational settings. Adequate water intake is crucial for preventing dehydration and fluid intake of 250–300 mL/hr in reducing dehydration. Regular rest breaks in shaded areas are essential for allowing workers to recover from heat exposure and prevent heat stress. These interventions work collectively by ensuring adequate hydration, allowing gradual acclimatization, providing regular cooling breaks, controlling environmental conditions, and ensuring early detection and treatment of heat-related symptoms (17, 18). The objective of this study is to enhance overall health and decrease susceptibility to extreme temperatures among outdoor workers. Additionally, it seeks to formulate comprehensive workplace and labour policies to prevent health risks for the large population of unorganized outdoor workers.

## 2 Physiology of heat stress

**Figure 1:**
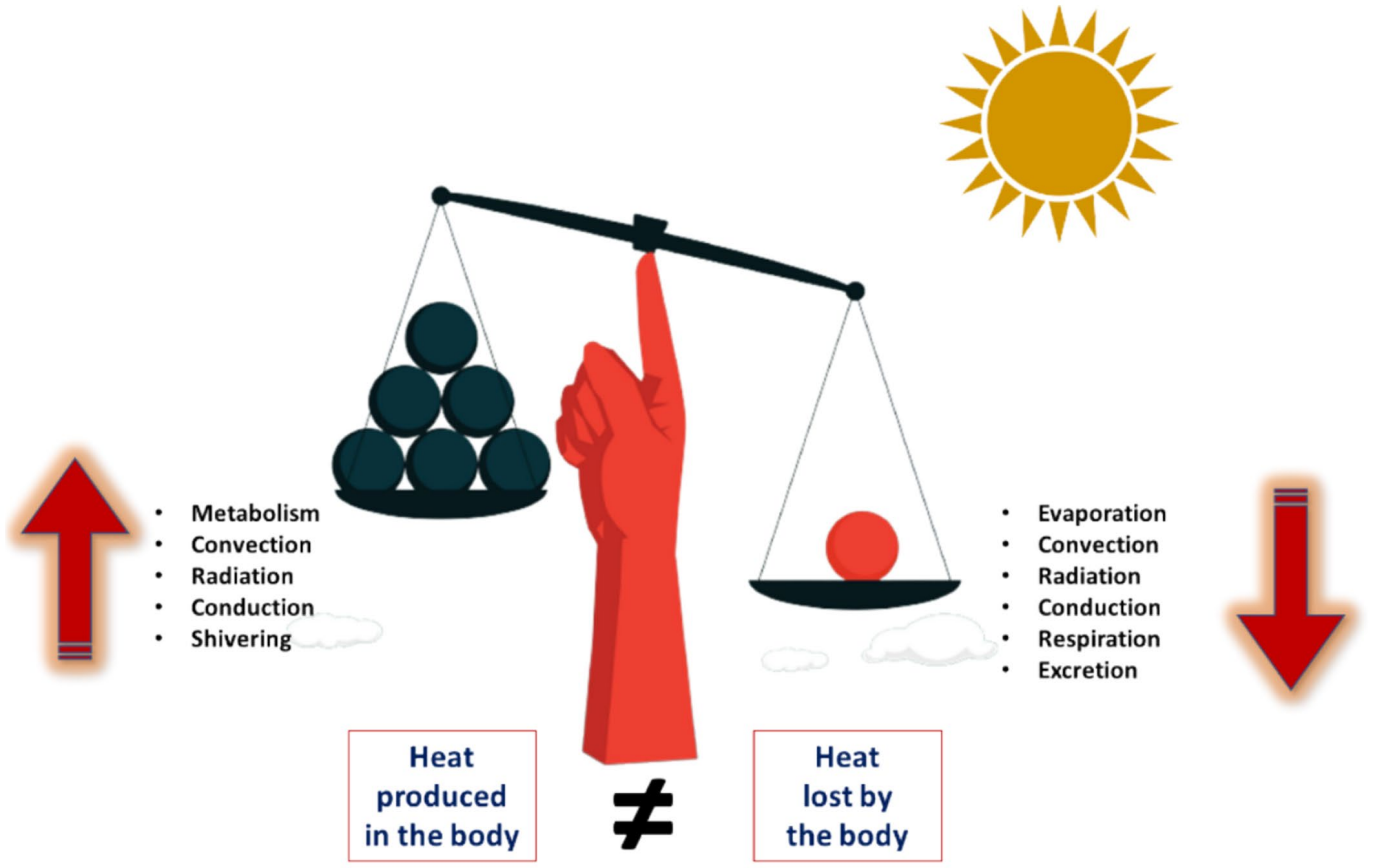
Human thermal imbalance.

Heat, as a fundamental form of energy, helps in optimal functioning of human body, typically maintains temperature approximately 36.6°C. The hypothalamus, acting as the central thermostat, regulates these processes by triggering responses such as sweating, shivering, and adjusting blood flow to maintain a stable internal temperature (19). To maintain this optimal temperature, the human body employs various mechanisms to regulate heat loss, either by promoting or reducing it (20). Heat loss mechanisms include radiation, conduction, convection, and evaporation. Environmental temperature, humidity, air movement, clothing, and body composition influence these processes. The body conserves heat through insulating mechanisms such as subcutaneous fat and hair and regulates heat loss through circulatory adjustments like vasodilation (increasing blood flow to the skin to lower heat) and vasoconstriction (decreasing blood flow to the skin to save heat). In response, the brain adjusts breathing rate, blood sugar levels, and metabolic rate to counterbalance temperature variations, triggering responses like shivering and sweating to maintain optimal body temperature. The imbalance in thermal equilibrium may leads to heat strain (see Figure 1).

Heat strain can be classified based on physical symptoms associated with heat, such as excessive thirst, fatigue, excessive sweating, and uncomfortable warmth. Exposure to high environmental temperatures leads to several physiological changes in the human body. The human body operates optimally within a specific temperature range, showcasing its remarkable biological design (21). When the environmental temperature exceeds the Core body temperature (CBT), the body begins to gain heat. The hypothalamus regulates CBT by triggering thermoregulatory mechanisms such as sweating and vasodilation to dissipate excess heat. An increased sweat rate (SwR) occurs when sweat glands activated by the sympathetic nervous system produce more sweat for evaporative cooling. This higher SwR can cause significant fluid and electrolyte loss, necessitating adequate hydration (22). Additionally, the metabolic rate and oxygen demand rise with higher temperatures, causing an increase in heart rate (HR) due to sympathetic activation and the need for enhanced blood flow through dilated peripheral vessels. Initially, heat exposure may cause a transient rise in blood pressure (BP) due to vasoconstriction, but prolonged exposure usually leads to vasodilation and a subsequent drop in BP, which can result in orthostatic hypotension. One of the indicators of dehydration is urine specific gravity (USG), when body becomes dehydrated due to heat stress, urine specific gravity increases, reflecting higher solute concentration due to reduced water content. Elevated USG values indicate that the kidneys are conserving water, which is a common physiological response to dehydration. Therefore, monitoring USG can be an effective way to assess hydration status and the impact of heat stress on the body. Heat-induced dehydration reduces blood volume, resulting in decreased blood flow and GFR and it leads to impair the kidney function. The body’s physiological response is to eliminate excess heat (23). The Heat Strain Index (HSI) is a quantitative measure to assess individuals exposed to high temperatures. It helps estimate the level of heat stress a worker may face based on the surrounding environmental factors and their physical activities (24). If it is prolonged which leads to heat-related illnesses (HRIs) may occur when the body cannot effectively cool itself, leading to conditions that range in severity from mild to life-threatening (25). HRIs requires immediate medical attention as it can become fatal if not treated earlier.

## 3 Methodology

This study aims to address several key research questions, to find out the heat stress levels among the workers and to see the effectiveness of “Water, Rest, Shade” (WRS) intervention in reducing these stressors. Additionally, it seeks to determine the most effective methods and target audiences for disseminating the study’s findings to encourage the widespread adoption of heat interventions, ultimately aiming to protect outdoor workers in the future.

### 3.1 Study settings

This is a quasi-experimental study designed to assess the effectiveness of the intervention which establishes a causal relationship between an intervention and its outcomes. Workers will be employed in outdoor occupations, such as agriculture and brick kilns.

### 3.2 Participant’s recruitment

Prior permission will be obtained from the relevant workplaces. Upon agreement to participate, 250 outdoor workers will be recruited, and informed consent will be obtained from each participant. The study will be explained to them in their preferred language to ensure understanding. The workers are included based on the criteria as follows: workers must be above the age of 18, have more than one to two years of experience in their current workplace, and be willing to participate in the study. Exclusion criteria for the study include workers over the age of 65, those with pre-existing illnesses, migrant workers, and individuals who plan to move out of the study area within the next 18 months.

### 3.3 Sample size calculation

**Figure 2:**
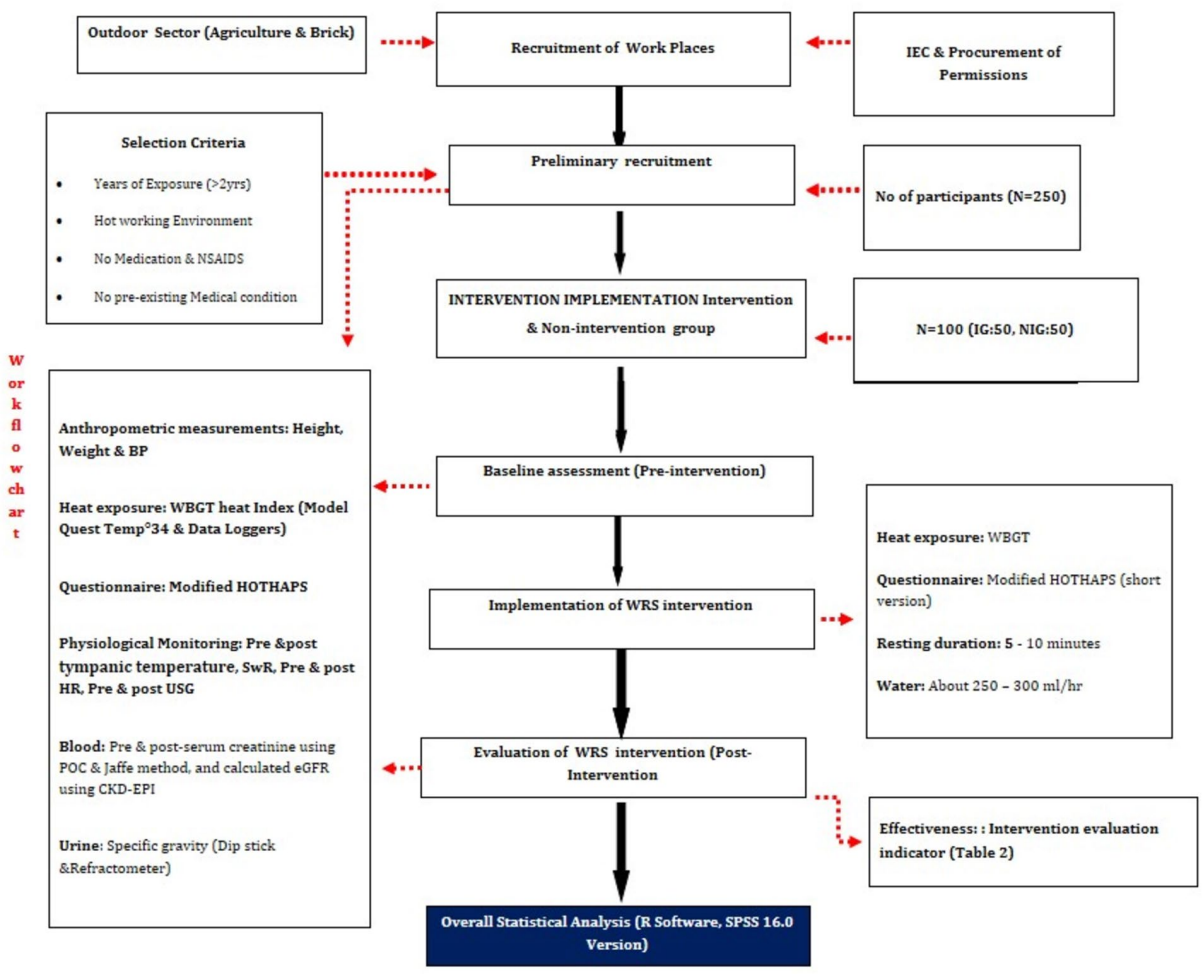
Work-flow chart.

Based on published literature, the sample size determination relied on the methodology outlined in the research article by (26) in Andhra Pradesh was taken for the sample size from outdoor workplaces in the defined study area. The study showed a prevalence of 6% of self-reported heat-related illnesses who has not taken the traditional diets and hence this was taken as the prevalence for the present study. Using the formula N = (Z^2^ * p * q)/d^2^, where Z is the alpha value for a 95% confidence interval (1.96), p is the prevalence rate (16%), q is 1 minus the prevalence, and d is the precision (4.5%), a sample size of 250 participants was determined for the overall population in the study area. For evaluating the effectiveness of the intervention, the 250 participants are separated into two groups the sample size was arrived at with the following scientific approach. Since there are not many studies linking heat stress and interventions, and one study has used the below-mentioned sample size (27). The formula used was n1 = n2 = (z_α_ + z_β_)^2^(p1(1 − p1) + p2(1 − p2))/(*δ* − ∣*ε*∣)^2^, where n₁ and n₂ represent the intervention and non-intervention group populations, respectively. Here, zα is 0.05, zβ is 0.20, p₁ is assumed to be 80%, p₂ is 95%, δ is 0.195, and ε is 15%. Based on this calculation, 50 participants were allocated to each group, allowing for a balanced evaluation of the intervention’s impact on heat-related illnesses (Figure 2).

### 3.4 Study procedure

The field data collection will occur over two summer seasons, involving four stages of field visits at a single location (Table 1). Firstly, the workers will undergo baseline assessments specific to outdoor work environments. Once the outdoor workers are selected, demographic details will be obtained, along with information about their work history and any prior similar roles. Data on additional physical activities (type of work, working hours, and break time) will also be gathered. Subsequently, informed consent will be secured from the workers after thoroughly explaining the study’s risks and benefits, along with their willingness to participate. A baseline investigation will be conducted, involving anthropometric measurements of height and weight. A walk-through survey will aid in selecting locations for profiling area heat exposure levels near the working place.

**Table 1:** Phases of data collection for one workplace during summer.

No	Particulars	Baseline intervention	Pre-intervention phase	Intervention phase	Post intervention phase
I	Number of field visits	1	1	1	1
	Duration	1 month	1 day	5 days	1 day
II	Participants	250	IG:50, NIG:50	IG:50, NIG:50	IG:50, NIG:50
III	Anthropometric data				
	Height	Yes	Yes	Yes	Yes
	Weight	Yes	Yes	Yes	Yes
	Physiological Monitoring: Pre & post tympanic temperature, SwR, Pre & post HR, Pre & post USG	Yes	Yes	Yes	Yes
IV	Modified HOTHAPS Questionnaire	Yes	Short version	Short version	Intervention Effectiveness questionnaire
V	Bio monitoring				
	Blood sample	Pre & post-serum creatinine using POC & Jaffe method, and calculated eGFR using CKD-EPI	Pre & post-serum creatinine using POC & Jaffe method, and calculated eGFR using CKD-EPI	-	Pre & post-serum creatinine using POC & Jaffe method, and calculated eGFR using CKD-EPI
	Urine sample	Specific gravity (dip stick & refractometer)	Specific gravity (dip stick & refractometer)	-	Specific gravity (dip stick & refractometer)
VI	Exposure				
	WBGT	Daily measured			
VII	Water refills	Daily self-report			
VIII	Difficulty of work	Daily self-report			

IG: Intervention group, NIG: Non-intervention group.

### 3.5 Exposure assessment

To assess workplace heat exposure, the Quest Temp 3 M WBGT Monitor (QuesTemp34; QUEST Technologies, United States) to measure environmental climatic conditions (ACGIH 2018) will be mounted in the workplace, and participants’ personal heat exposure will be continuously monitored using Easy Log data loggers (EL-USB-2-LCD+ model), data loggers automatically track and log environmental variables over time, making it possible to measure and record conditions.

### 3.6 Physiological heat strain indicators

The heat strain experienced by the workers will be assessed through selective physiological responses, including pre- and post-shift tympanic temperatures, urine specific gravity (USG), sweat rate (SwR), and heart rates (HRs) to evaluate hydration status and heat strain.

### 3.7 Blood samples

A 2–3 mL sample of blood in vaccutainers (Becton Dickinson, Franklin Lakes, and, NJ) and send it to the laboratory for eGFR calculation using CKD-EPI. An indicator of kidney health, the estimated glomerular filtration rate (eGFR) calculates how well the kidneys filter blood (28). The serum creatinine blood test measures the level of creatinine in the blood using a point-of-care device.

### 3.8 Urine samples

Onsite urine assessments (USG, pH, leucocytes, hematuria, and proteinuria) will be conducted using a dipstick and refractometer. A refractometer is a device that measures the extent to which light bends when it moves from air into a sample that measures the USG it is a proxy measure to identify the level of hydration. Normal USG values range from 1.002 to 1.019. Using a sample container, a 5-ml urine sample will be taken. Samples will be kept in the field for up to six hours in ice boxes kept at 4°C before being sent to the collaborating lab for analysis.

### 3.9 Perception data

During lunch, workers will be administered the modified version of the “High Occupational Temperature Health and Productivity Suppression” (HOTHAPS) questionnaire. It will be used to gather information on symptoms of heat stress and heat strain, including urogenital problems. The purpose of the questionnaire is to help assess how workers are affected by heat exposure. Additionally, how the heat affects their daily activities and health issues, and problems associated with working in hot conditions will be collected.

### 3.10 Ethics considerations

Informed consent will be obtained from all participants before their involvement in the study. The study’s objectives, procedures, benefit, potential risks, and their right to withdraw from the study at any time will be explained to them. Access to data will be limited to authorized personnel only, and results will be reported in aggregate to prevent identification of individuals. Ethical approval for the study has been obtained from the Institutional Ethical Committee of Sri Ramachandra Institute of Higher Education and Research, ensuring ethical guidelines.

### 3.11 Data management plan

The data management plan involves data integrity and security. Physiological measurements, including tympanic temperature and heart rate, will be recorded according to standardized protocols, and laboratory analyses will be conducted using validated methods. Data from the modified HOTHAPS questionnaire will also be collected. All data will be stored in password protected electronic files and with regular backups to prevent data loss. Access to the raw data will be restricted to authorized research team members, Statistical software will be used for data analysis, following established guidelines to ensure accuracy and validity. Findings will be shared through peer-reviewed publications and presentation. According to institutional and ethical guidelines, the data will be stored upto 10 years after completion of project. During this period, the data may be used for potential reanalysis or follow-up of any other studies. After 10 years, the data will be securely archived.

Pre-shift assessments will include anthropometric assessments, exposure assessments, physiological heat strain indicators, urine samples, and the HOTHAPS questionnaire. At mid-shift. Assess workers weight, collect urine samples, and record their water consumption and collect urine sample. The difference in pre-shift and mid-shift body weight and fluid consumption is divided by the observation time to get the SwR. Then monitor physiological heat strain measurements post-shift, right before they take a rest at the end of their shift. Collected post-tympanic temperature, post-HR, post-BP, and post-USG readings, take blood samples, and conduct point-of-care serum creatinine tests. Assessed urine samples using urine dipsticks and a USG refractometer.

### 3.12 Pre—intervention phase

After the baseline investigations, which include all 250 participants, the workers will be divided into two groups. They are matching based on participant’s age, gender, education, workload, and other exposures (smoking, alcohol, and duration of employment) and 50 workers will be on the intervention group, 50 workers in the non-intervention group. The participants will be selected based on various factors, including age, gender, workload, education level, as well as other relevant exposures such as smoking, drinking habits, and length of employment. By considering these variables, Our aim to minimize any potential confounding factors and enhance the validity of our research findings. Again, the pre-shift and post-shift assessments and HOTHAPS questionnaire will be administered for both groups.

### 3.13 Intervention phase

A heat stress intervention consisting of WRS will be provided to the intervention group (*N* = 50) daily throughout the entire day for a duration of one week. This will include a fluid intake of about 250–300 mL/h, along with regular breaks and rest in a shaded area every 5–10 min.

### 3.14 Post-intervention phase

After the intervention is administered, baseline assessments will be repeated for both groups to see the effectiveness, and the interventions will be assessed based on the outcome indicators shown in Table 2. The tympanic temperatureshould not increase more than 10°C, and the Sweat rate should not increase by 1 litre per hour. There should be a decrease in urine specific gravity (USG), heart rate, and creatinine levels after receiving the WRS intervention.

**Table 2:** Intervention evaluation indicator.

S. no	Evaluation indicators		
1	Heat strain indicators	Tympanic temperature (Ttemp)	No increase from baseline
		Sweat rate (SwR)	No increase from baseline
		Urine specific gravity (USG)	Decrease from base line
		Heart Rate	Decrease from base line
2	Indices of kidney function	Blood parameters	Creatinine mg/dL (seasonal): Decrease from baseline Creatinine mg/dL (cross-shift for Acute Kidney Injury): Decrease
3	Urine parameters	Dipstick	pH: <6

### 3.15 Statistical analysis

Data entry and consolidation will be done in Microsoft Excel, while the statistical analysis will be carried out using SPSS version 16.1. Calculations will be performed to analyse the descriptive statistics from demographics, heat exposure, health symptoms, and physiological indicators. The dependent variables will be classified based on the presence or absence of symptoms related to heat strain, dehydration, and urogenital issues. Physiological indicators like tympanic temperature, SwR, USG, and eGFR will be classified based on the standard range. Analyse categorical variables using chi-square test.

Use the Wilcoxon signed rank test to compare the levels of continuous variables before and after a shift, including physiological heat strain signals like pH, hematuria, and proteinuria. A multivariate logistic regression (MLR) model will be utilised to systematically adjust for confounding factors. Prior to conducting the MLR, To ensure that all confounding factors to be included in the analysis have been finalized. Given the interdependence of age and years of exposure, a correlation analysis will be performed using the Spearman correlation test to examine the relationship between these two variables. Considered several important factors, such as age, gender, years of exposure, and education, which could potentially influence the results. After finalizing the confounders, we will proceed with the MLR. In the initial step, Perform a chi-square test to ascertain the association between the dependent and independent variables. During the second stage, Do calculations to find out the association of the odds ratio (AOR) between the independent and dependent variables. Take into account important confounding variables and exclude any confounders that are not statistically significant.

## 4 Challenges and feasibilities in conducting WRS interventions in LMICs like India

There are only a few studies done on giving water, rest, and shade (WRS) heat interventions to outdoor workers (16–18, 29, 30). Table 3 summarizes different workplace interventions aimed at ensuring workers stay hydrated and get adequate rest to prevent HRIs in developed countries. Each intervention aims to balance hydration and rest to ensure worker safety and efficiency, with slight variations in how water is provided, how much water is consumed, and the frequency and duration of rest breaks. Conducting WRS interventions in LMICs like India differs significantly from those previously done in Central American studies due to factors such as workplace infrastructure, worker demographics, climate conditions, and cultural and economic factors. Workplaces in the developed countries often have better infrastructure, systematic rules regulations and policies and workplace heat standards and advisories to implement to ensure that workers have access to shade, water, and appropriate rest breaks during heat events (17). Additionally, initiatives like green infrastructure projects, which include urban greening and cool roofs, are promoted to mitigate the heat-island effect and provide cooler working environments, facilitating easier implementation of shaded rest areas and access to large quantities of water (29). Whereas Indian sites may lack these facilities, which need a more tailored approach. Even though, India has few regulations formulated recently (31) to improve workplace and workers conditions to prevent HRIs, the awareness and enforcement is being inconsistent, affecting the implementation of safety protocols. Furthermore, the behavioral practices towards rest breaks and hydration vary, with Indian workers often resist to frequency in the rest breaks due to productivity and economic concerns. By understanding and addressing these differences, our study aims to provide a practical and effective trial model for implementing WRS interventions in outdoor workplaces, ensuring worker safety and productivity based upon our own pilot study (unpublished results).

**Table 3:** Previous studies on water, rest and shade (WRS) intervention.

Author Name	Year	Country	Heat stress intervention			Outcome
			Water	Rest and shade	Others	
Bodin et al.(17)	2016	Central America	3 litres water bag and refill every break attent	10–15 min rest breaks at tent	Redesigned machete	The production had a significant rise, rising from 5.1 to 7.3 tonnes per person per day after the intervention and Heat stress and dehydration were reduced.
Butler-Dawson et al. (29)	2019	Central America	Encouraged Workers to drink additional water, Electrolyte solution and take more rest breaks.		Education program on the importance of WRS	Hydration is important and protective
Wegman et al. (18)	2018	Central America	3-liter water backpack, 40 L water to refill	10–15-min rest	lighter machete with curved blade	Impact of heat stress are reduced
Glaser et al. (16)	2022	Central America	5 litres water bag, 300 mL of electrolyte solution from a tent.	20 min of rest every hour and consume	Nil	Intervention evolved over time, including improvements in changing of WRS. The impact of these improvements on mitigating heat stress is also assessed.
Hansson et al. (30)	2024	Central America	Both water and rehydration solution	20 Minutes Rest	Nil	Productivity increased during the study period.

## 5 Our own pilot study conducted in India

After considering the above challenges conducted a pilot study with 50 participants Intervention group has 25 participants and 25 in non-intervention group and implemented a one-week intervention providing 250–300 mL of water and 5 to 10 min of rest every hour, providing rest for more than 10 min disrupts the workflow, as restarting work can be challenging and employers are not willing to give a break to workers every hour due to the productivity loss. To address this, compensated the workers for the given rest, time and managed the rest periods by staggering the breaks. Then, Divided the 25 participants in the intervention group into 5 batches, with each batch taking a rest at different times. This staggered approach ensured that the entire group did not rest simultaneously, maintaining workflow continuity. The perception results showed that there was a significant reduction in prevalence of heat stress symptoms, especially excessive thirst, excessive sweating, tiredness, and prickly heat after intervention (76% has reduced to 24%). The non-intervention group had 10 times greater odds of adverse health outcomes than the intervention group. The results showed a significant difference in reduction in CBT, with the non-intervention group being 12 times more likely to have increased CBT (unpublished result). By understanding and addressing these differences, our study aims to provide a practical and effective trial model for implementing WRS interventions in outdoor workplaces, ensuring worker safety and productivity.

## 6 Limitations

Separating the outdoor workers into two distinct groups may lead to selection bias, potentially affecting the general stability of the results. To manage this, matched participants in the intervention and non-intervention groups based on demographic and work-related characteristics to reduce bias and increase result stability. Due to the practical constraints of fieldwork, it is unable to include continuous core body temperature monitoring, which is considered the gold standard. Instead, tympanic temperature will be used as a measure of core temperature. Self-reported data through the HOTHAPS questionnaire may also introduce recall bias, participants may under report or over report symptoms and perceptions of heat stress.

Working in extreme heat conditions leads to several challenges, particularly in ensuring that all participants fully understood the study procedures and provided informed consent. Some workers, especially those from migrant backgrounds, were initially hesitant to share personal information, complicating the consent process. Additionally, difficulties in reaching all eligible workers due to their demanding work schedules and family commitments limited the study’s sample size.

Conducting field under scorching heat leads to physical and mental effects on the research team. Prolonged exposure to high heat, leads to fatigue and exhaustion. This fatigue resulted in measurement errors, incomplete interview transcripts, and delays in reporting. Efforts were made to mitigate fatigue through regular breaks and hydration.

Limited outdoor workplace infrastructure, including inadequate sanitation had a impact on research team’s efficiency and well-being. Poor sanitation facilities increased the risk of illness, directly affecting field productivity.

## 7 Expected outcome

The primary goal of the “Water, Rest, Shade” (WRS) intervention is to implement a practical and effective strategy for managing heat stress in outdoor workplaces. The intervention is anticipated to bring several positive outcomes. Firstly, a significant reduction in heat stress among the intervention group, as indicated by improvements in physiological measures such as tympanic temperature, heart rate, sweat rate, and urine specific gravity. Additionally, the intervention group reported a decrease in the incidence of heat-related health issues, such as dehydration and heat exhaustion, and a reduction in self-reported heat-related health symptoms, as measured by the modified HOTHAPS questionnaire.

The intervention will lead to enhanced overall well-being for outdoor workers. Increased comfort, a decrease in heat-related illnesses, and potentially fewer work-related accidents are likely to reflect this improvement. We anticipate that the intervention, by effectively managing heat stress, will boost productivity and reduce absenteeism, thereby benefiting workers.

The study will also aim to identify successful adaptation techniques for implementing heat stress interventions in outdoor settings. These insights will contribute to developing better strategies for reducing heat vulnerability and improving workers’ quality of life. Finally, we expect the findings to support the development of comprehensive heat stress management policies and practices, providing valuable evidence for the development of future interventions and guidelines for similar occupational environments.

## 8 Conclusion

This study will demonstrate the effectiveness of the intervention and offer valuable strategies for mitigating heat stress. It will focus on the successful implementation and assessment of both health impacts and implementation quality and provides a fresh perspectives on the importance of this intervention in combating scorching heat. Additionally, it will help in the creation of inclusive workplaces and labour policies that focus on reducing health hazards for the large population of unorganised outdoor workers. The study also aims to provide crucial information to raise awareness about the importance of preventing heat stress among workers, especially those who work outdoors. This information will be helpful in developing comprehensive workplace and labour policies to protect the health of millions of unorganised outdoor workers.

## 9 Dissemination

After the study is completed, the results will undergo analysis and be prepared for publication. After the project concludes, a community event will be organized to share the findings and raise awareness among the workers took part in the study.

## Data Availability

The original contributions presented in the study are included in the article/supplementary material, further inquiries can be directed to the corresponding author.
